# Emerging Roles, Mechanisms, and Therapeutic Potential of Thyroid Hormones in Neurodegenerative Diseases: A Review

**DOI:** 10.3390/brainsci16020229

**Published:** 2026-02-14

**Authors:** Xin’ai Li, Zhe Li, Manna Sun, Yunlong Du, Han Bai, Xiaoheng Chen, Junhui Wang

**Affiliations:** 1 Department of Thyropathy, Dongzhimen Hospital, Beijing University of Chinese Medicine, Beijing 100700, China; 2 Department of Clinical Laboratory, Lixing Traditional Chinese Medicine Hospital, Kaifeng 475400, China; 3 The 3st Ward, Department of Cardiology, Tongxu No.1 Hospital, Kaifeng 475400, China; 4 Thyropathy Hospital, Sun Simiao Hospital, Beijing University of Chinese Medicine, Tongchuan 727000, China; 5 Lunenfeld-Tanenbaum Research Institute, Mount Sinai Hospital, Toronto, ON M5G 1X5, Canada

**Keywords:** thyroid hormones, neurodegenerative diseases, Alzheimer’s disease, Parkinson’s disease, thyrotropin-releasing hormone, oxidative stress, neuroprotection, therapeutic potential

## Abstract

Thyroid hormones (THs) are master controllers in the endocrine system and have drawn considerable attention from the research community due to their associations with neurodegenerative diseases as well. In this review article, we present a comprehensive summary of the physiological functions and pathogenic mechanisms of THs in the regulation of several representative neurodegenerative diseases. Our study particularly focuses on Alzheimer’s disease (AD), Parkinson’s disease (PD), and multiple sclerosis (MS). AD is the most common cause of dementia, primarily caused by tau protein tangles inside nerve cells and β-amyloid plaques outside, which lead to nerve cell death and brain atrophy. PD is primarily a movement disorder. The degeneration of dopaminergic neurons in the brain impairs the brain’s control over muscle activity. MS is usually considered to be an autoimmune demyelinating disease, but it has been found that MS also presents with secondary neurodegenerative pathology, including axonal loss and neuronal damage. In this review, the effects of TH on the pathogeneses of AD, PD, and MS are discussed in detail, with a focus on the following potential mechanisms: neuroprotection, neurogenesis, oxidative stress, and inflammatory response. In addition, we conduct an in-depth review of the possible clinical applications of TH, TH analogs, and thyrotropin-releasing hormone (TRH) in the treatment of AD, PD, and MS based on recent preclinical and clinical studies. By integrating experimental, clinical, and epidemiological results on the effects of TH on neurodegeneration, the present review constructs a theoretical basis for the involvement of TH in the pathogeneses of these diseases in detail. We believe that this basis will be useful for clinical diagnosis and treatment.

## 1. Introduction

Thyroid hormones (THs) are endocrine regulatory molecules secreted by the thyroid gland, which are critical for the body’s metabolism and growth and development. They are iodinated tyrosine derivatives, mainly including two active forms, thyroxine (T4) and triiodothyronine (T3) [1,2]. They belong to iodinated aromatic amino acid derivatives. Under physiological conditions, they exist as zwitterions and exhibit lipophilicity, a property that influences their distribution in the body [3]. In the blood, they primarily bind to specific plasma proteins for transport and exhibit high affinity for nuclear receptors [4]. THs are widely involved in the regulation of various physiological processes such as metabolism, growth, development, and the functioning of the central nervous system (CNS). A vast body of literature has reported their important roles in brain development and protection of neuronal integrity. Among the CNS, THs modulate the expression profile of genes involved in neurogenesis, myelination, and synaptogenesis, which are responsible for the proper assembly and functioning of neuronal circuits [5]. The cerebrospinal fluid (CSF), which bathes the brain and spinal cord, contains unbound and transthyretin-bound TH—involved in brain maturation and metabolism, as well as other metabolic THs [5]. In the CNS, THs are involved in energy metabolism and thermogenesis through modulation of the processes of cellular respiration and mitochondrial function, which differ from those observed in peripheral tissues [6]. Moreover, the hypothalamus–pituitary–thyroid (HPT) axis is responsible for the rigorous control of systemic TH levels, which is regulated by feedback inhibition of thyroid-stimulating hormone (TSH) and TRH secretion from the hypothalamus [7].

The thyroid hormone (TH) signaling network relies on transmembrane transport and deiodinase-mediated local metabolism (converting T4 to active T3) to regulate intracellular hormone levels [8,9,10]. Its mechanism of action encompasses genomic pathways—where T3 binds to tissue-specific nuclear receptor subtypes (TRα and TRβ) to regulate gene transcription, as well as transcription-independent rapid signaling cascades. The integration of this dual mechanism precisely maintains the body’s development, energy metabolism, and systemic homeostasis [11,12,13].

Thyroid hormone receptors (TRs) are essential for maintaining metabolic homeostasis and modulate the functioning of different organ systems, including the CNS [7]. TRs, which are ligand-dependent transcription factors, mediate most of TH’s actions and play a crucial role in maintaining metabolic homeostasis while broadly regulating the functions of various organ systems, including the CNS [14,15]. TRs exhibit a complex expression pattern in the CNS. They play a central role in many key brain functions such as neurodevelopment, myelination, energy homeostasis, behavioral plasticity, and circadian rhythms through regulation of gene transcription. Dysregulation of their signaling pathways is associated with various neurodevelopmental disorders, mental illnesses, and demyelinating lesions, and agonists targeting specific TR subtypes have emerged as a promising new strategy for treating metabolic, genetic, and inflammatory neurological diseases [16,17,18].

Therefore, the relationship between THs and the function of the CNS further demonstrate the important role of these hormones on neuronal plasticity and neurotransmission as well as oxidative phosphorylation, which are required for the proper states of cognition and emotion [19].

Neurodegenerative diseases (NDDs) are marked by a decline in neuronal integrity that includes several pathologic conditions such as oxidative stress, neuroinflammation, mitochondrial dysfunction, protein misfolding, and neuronal apoptosis, which lead to the decrement of cognitive and motor functions in AD, PD, Huntington’s disease, and MS [20,21]. The CNS is particularly vulnerable to defects in oxidative phosphorylation and mitochondrial function, which are aggravated by aging and diseases, finally compromising neuronal survival and synaptic integrity [19].

Thyroid hormone (TH) modulates the different underlying pathogenic mechanisms. Any increase or decrease in TH levels can induce defects in neurotransmission, increased oxidative stress, and altered glucose metabolism in the brain, potentially leading to cognitive deficits and acceleration of the neurodegenerative process [22,23]. The HPT axis is closely related to neuronal systems, and in PD, the abnormal regulation of TH has been related to the severity and progression of the disease, probably due to the involvement of similar genetic and molecular traits with PD and thyroid disorders [24]. These features include mitochondrial dysfunction, elevated oxidative stress levels, and abnormal glucose metabolism, which play crucial roles in the neurodegenerative pathology of PD [25,26]. Specifically, the pathogenesis of PD significantly overlaps with age-related biological changes, such as impaired mitochondrial function and oxidative stress, which are also hallmarks of cellular senescence [25]. Meanwhile, PD patients exhibit varying degrees of disorders of glucose metabolism at different stages of the disease, involving multiple key processes such as glycolysis and the tricarboxylic acid cycle [26]. Deficiencies in these cellular metabolic functions are considered key factors contributing to neuronal death and abnormal immune-inflammatory responses in PD [27]. Since THs are core hormones regulating mitochondrial function, redox balance, and glucose metabolism, genetic variations or molecular dysregulations modulating these common pathways may simultaneously influence susceptibility to PD and thyroid diseases, as well as their progression, constituting a similar molecular basis for both [26]. There is a feedback loop between thyroid dysfunction and neurodegeneration. Variability in the signaling of THs can contribute to the onset of NDDs and, conversely, the emergence of NDDs can modulate TH signaling with a vicious circle that promotes disease progression [23]. In addition, neuroendocrine components, such as neuropeptides and hormone receptors, play important roles in neuroprotection and neuronal survival. Therefore, the roles of TH in the pathogenesis of CNS disorders need to be elucidated [28,29].

In recent years, abnormal thyroid hormone levels have been connected with neurodegenerative disorders. Hypothyroidism and hyperthyroidism are associated with cognitive decline and changes in the levels of biomarkers, such as amyloid-β(Aβ) peptides and tau proteins [23,30]. The validity of these findings is further supported by animal studies. For example, changes in the levels of TH, which can happen during development or be induced in animals, lead to lifelong deficits in glial function, which in turn cause enduring impairments in neurogenesis and cognition [31]. In addition, THR and TRs exhibit distinct expression patterns in the brain, which implies that the specific roles of thyroid hormone receptor isoforms in neuronal development and metabolism exist and might profoundly affect the vulnerability to different diseases and the effectiveness of treatments [29,32]. Sobetirome, a thyroid hormone analog, and its CNS-targeting prodrugs, which are selective TRβ agonists, exhibit great potential in promoting remyelination and altering neurodegenerative pathology in preclinical studies [16]. Moreover, environmental toxins and pharmacological agents affect thyroid hormone transport and metabolism. This disruption provides further evidence for the complexity of maintaining thyroid hormone homeostasis in the CNS and its consequences for health [33,34]. Taken together, these advances indicate the increasing awareness of TH and their signaling pathways as critical components in the pathogenesis of NDs and as potential targets for new therapies. This review summarizes recent advances in the field of TH’ roles in NDs. It discusses their mechanisms, effects on pathogenic processes, signaling pathways, receptor isoforms, and therapies with the aim of bridging research and clinical applications for new treatments.

## 2. The Biosynthesis of TH and HP

The synthesis of TH is a complex process that requires iodine and is mediated by thyroglobulin (Tg). This process is controlled by the hypothalamic–pituitary–thyroid (HPT) axis through positive and negative feedback mechanisms such as TRH and TSH. In addition, the iodide transport into the thyroid gland through the sodium-iodide symporter, or the oxidation of iodide and the subsequent incorporation into Tg by thyroid peroxidase and this process need the hydrogen peroxide synthesized by dual oxidase. Finally, iodinated Tg is subjected to proteolytic cleavage to release T4 and T3 into the bloodstream. The regulation of thyroid hormone synthesis is characterized by feed-forward and feedback mechanisms that include the Wolff–Chaikoff effect, which aims to reduce hormone production when there is excess iodide (TG feedback) and, in turn, feedback from TSH receptor [35,36]. The HPT axis stimulates the pituitary gland to release TSH through the secretion of TRH from the hypothalamus, thereby promoting the synthesis and secretion of thyroid hormones (T4 and T3) by the thyroid gland. The T4 and T3 in circulation regulate the secretion of TRH and TSH through negative feedback, maintaining hormonal balance. When the level of thyroid hormones decreases, the production of TRH and TSH increases; conversely, it is inhibited. Other factors can also regulate this axis [37]. Transcription factors PAX8 and NKX2.1 play a key role in the regulation of expression of the Tg gene. On the other hand, post-translational modifications and transport steps are responsible for proper folding and functioning of Tg and other proteins involved in hormone synthesis. The chaperone protein GRP170 is located in the endoplasmic reticulum and is crucial for the maturation of the TSH receptor. Its absence may be associated with improper folding of the receptor, thereby reducing the reactivity of the thyroid [36] https://pubmed.ncbi.nlm.nih.gov/25146893/, accessed on 1 November 2025. Moreover, hydrogen sulfide (H2S) generated by cystathionine β-synthase (CBS) situated in thyrocytes, enhances thyroid hormone synthesis by increasing the level of sirtuin-1 (SIRT1), which in turn enhances expression of other key proteins, such as TPO, NIS, Pendrin or MCT8. Thus, gasotransmitters also play a very important role in thyroid function [38]. Environmental pollutants, such as bisphenol derivatives or per- and polyfluoroalkyl substances (PFAS), induce disturbances in thyroid hormone production through improper functioning of the iodide absorption process, iodination of Tg or synthesis of T4. Particularly, excess of iodide leads to activation of XBP1 and unfolded protein response, which negatively influences thyroid function, especially in case of iodine deficiency [39]. HPT axis regulates the release of TSH from pituitary gland, through feedback from TH concentration and models simulating the TSH variations and cross-relationships are important for the process of maintenance of homeostasis.

**Figure 1:**
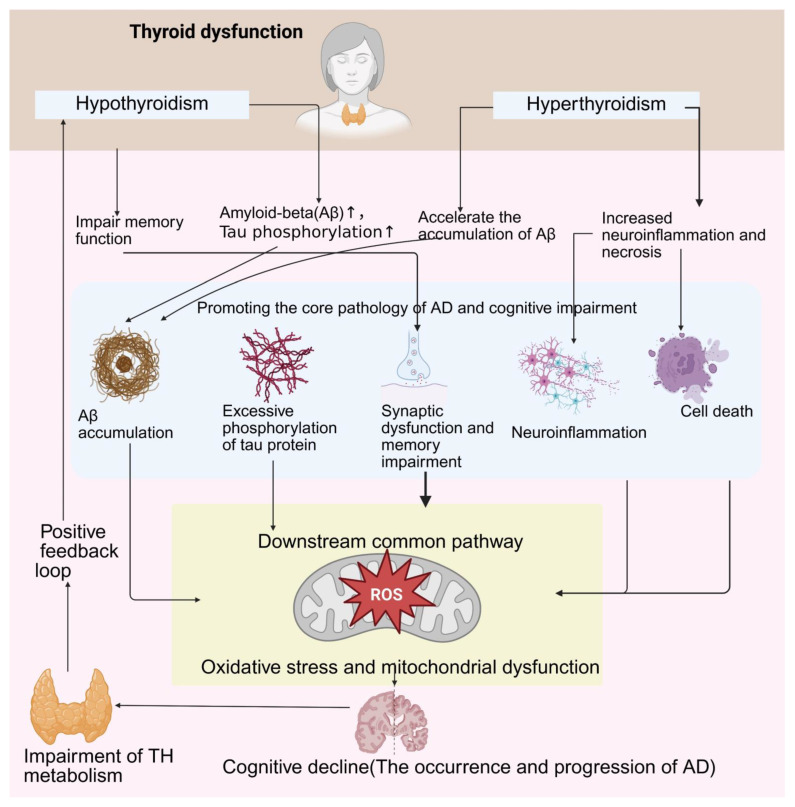
Schematic diagram of thyroid hormone synthesis, secretion and regulatory mechanisms. (PAX8: Paired Box Gene 8; Nrf2: Nuclear factor erythroid 2-related factor 2; NIS: Sodium-Iodide Symporter; TPO: Thyroid Peroxidase; TG: Thyroglobulin).

The thyroid gland has autonomous regulation of hormone output through both feed-forward and feedback regulation, irrespective of the pituitary gland, and this regulation is elaborate. Thyroid follicular cells are adaptive, as shown in models of congenital hypothyroidism which demonstrated that follicular cells can survive ER stress-induced apoptosis. Thus, thyroid cells maintain some T4 production through unknown means involving iodinated Tg released from lysed thyrocytes within the follicular lumen [40]. Newly emerged ex vivo and in vitro techniques such as three-dimensional thyroid organoids and Transwell cultures using primary human thyrocytes have greatly improved thyroid differentiation and hormone production studies, greatly resembling the in vivo tissue environment. Nrf2 signaling pathway is involved in the antioxidant defense of the thyroid and in the processing of Tg, maintaining thyroid function under oxidative stress: redox homeostasis and hormone synthesis are linked [41]. Production and secretion of TH are meticulously regulated by an interplay involving iodine metabolism, production of Tg and various activities, and feedback signals from the HPT axis. These processes are modulated by intrinsic thyroid regulation and other hormones as well as environmental effects. The discovery of new pathways such as Nrf2 demonstrates the complexity of thyroid regulation; it also reminds us of the importance of understanding these processes to guide further studies toward developing new therapies for thyroid diseases as well as the impact of environmental factors on thyroid health [42] (Figure 1).

## 3. The Physiological Roles of TH and HPT

Thyroid hormones (THs) and the hypothalamic–pituitary–thyroid (HPT) axis have extensive and crucial physiological functions [37]. Thyroid hormones are essential for the development of the nervous system, linear growth, energy metabolism, and thermogenesis [37]. Metabolically, they regulate the liver’s nutrient metabolism, fluid balance, and cardiovascular system functions. Specifically, thyroid hormones are vital for maintaining metabolic balance in the liver and systemic metabolic balance [43]. Dysregulation of the HPT axis can lead to hepatic metabolic disorders, affecting lipid metabolism, glucose regulation, and protein synthesis. Reduced circulating and intrahepatic thyroid hormone concentrations can increase the risk of metabolic dysfunction-related fatty liver diseases by inducing lipotoxicity, inflammation, and fibrosis [43]. At the cellular level, the effects of thyroid hormones are primarily mediated by nuclear TRs, achieved by altering gene expression. T3 is the preferred ligand for THR. T4, which is approximately 100 times more concentrated in serum, is activated through conversion to T3 by 5′-deiodinase outside the thyroid. The regulation of deiodinases and thyroid hormone transporters on cell membranes, particularly the control of T3 availability, is fundamental to the action of thyroid hormones. Furthermore, studies have found that thyroid hormones may serve as a temporal cue for the central biological clock, with hypothyroidism altering the expression of clock genes in the suprachiasmatic nucleus, body temperature, and metabolic rhythmicity [44].

## 4. Thyroid Hormones and the HPT Axis in Neurodevelopment

### 4.1. The Roles of TH and HPT in Neurodevelopment

Thyroid hormones (THs), consisting of T4 and T3, are crucial for brain development and neuronal function. THs are involved in neuronal differentiation, migration, synapse formation, and myelination of nerve fibers. The timing and quantity of thyroid hormone availability are important because both hypothyroidism and hyperthyroidism during certain periods of development can result in severe neurological deficits [45,46,47]. THs act through genomic pathways involving nuclear TRs as well as through non-genomic actions associated with interactions with the neuronal plasma membrane to regulate the proliferation, maturation, and metabolic activities of neural progenitor cells [48,49,50].

The action of TH in the CNS is also modulated by transport proteins and deiodinases that affect the availability and movement of THs in different brain regions [51,52]. Variants in the genes encoding transporters and enzymes involved in thyroid hormone metabolism can lead to defects in myelination and oligodendrocyte development and may cause lifelong neurological impairments (e.g., in syndromes caused by MCT8 deficiency) [52]. The pattern of spatial and temporal expression of these factors makes the localized action of TH signaling particularly important.

Multiple in vitro studies have shown that environmental pollutants can interfere with thyroid hormone signaling and adversely affect gene expression and brain development [53,54,55,56]. Such observations support the idea that the developing brain is susceptible to changes in thyroid hormone levels and that THs play an important role in brain development, including the processes of dendritic arborization, axonal growth, and myelination at the neuronal level. At the cellular level, THs exert significant effects on neuronal function by modulating the properties of synapses and synaptic plasticity. In a more mature brain, THs affect both the excitatory and inhibitory components of synaptic activity, particularly in cortical circuits, thereby influencing cognitive functions and behaviors [57,58]. THs also promote maturation of some neuronal subtypes, such as GABAergic interneurons, which may help to establish inhibitory tone and overall stability of neuronal circuits [59]. In addition, THs modulate the expression of neurotrophic factors, such as brain-derived neurotrophic factor (BDNF), which is involved in processes of learning and memory, thus linking thyroid function to cognitive outcomes [60].

Thyroid hormone (TH) and other metabolic factors influence neuronal development. Low iron levels affect TH-regulated gene expression in hippocampal neurons, indicating their interdependence and the requirement for their coordinated regulation in energy metabolism and neuronal development [61]. Furthermore, THs are essential for the proliferation and functional activities of hypothalamic neurons that mediate energy balance and feeding behavior. This indicates their key role in the relationship between the endocrine and nervous systems [62]. THs are essential for brain development and function, they stimulate the growth and myelination of neurons, their actions are modulated by transporters and deiodinases; any abnormalities in these processes can lead to neurodevelopmental disorders, so it is important that appropriate levels of these hormones are maintained and suitable therapies found [46,52,53,57,62].

### 4.2. TH Receptors and Their Signal Transduction Pathways

Thyroid hormone (TH) enter the target cell and mediate their physiologic effects through genomic mediated by TH receptors, TRs, which bind to DNA and regulate gene transcription, essential for the development of the nervous system and the correct functioning of the mature brain [63,64]. Corepressors such as NCOR1 and NCOR2 repress gene expression in the absence of hormones; however, when hormones are present, coactivators such as SRC-1 enhance gene expression and fine-tune gene expression through histone modification [65]. Importantly, mutations in the TR genes lead to TH resistance syndrome [66,67].

Thyroid hormone (TH) also mediate rapid non-genomic signaling through integrin αvβ3 receptor, which modulates the intracellular signaling pathways such as MAPK/ERK1/2 and PI3K/AKT, which are crucial for the regulation of cell proliferation, survival, angiogenesis, and metabolism, playing an important role in neuroprotection after the cerebral injuries [68,69,70,71]. For example, T4 binding and signaling through integrin αvβ3 lead to the activation of MAPK/ERK1/2 signaling cascade, which plays a pro-survival role in neurons and modulates the expression of genes involved in the processes of oncogenesis and NDDs [72,73]. Therefore, environmental pollutants with TH activity (such as TDCPP), which could enter the cell and bind to integrin αvβ3 receptor, could modulate the intracellular signaling pathways described above and induce neurodevelopmental toxicity [74,75].

G protein-coupled receptors (GPCRs) transmit cellular signals in response to diverse stimuli, including thyroid stimulating hormone receptor (TSHR), which is involved in TH signaling and activates G protein pathways, which modulate cell growth and differentiation in thyroid and in brain [76,77]. Notably, signaling of TSHR is linked to specific cognitive functions and the preservation of synaptic structures since cognitive deficits were observed in TSHR-knockout mice and therefore is important for neuronal health. Moreover, the idea of compartmentalized GPCR signaling suggests that GPCRs could send signals from different intracellular compartments, such as endosomes and Golgi apparatus, which adds another level of complexity to the understanding of the mechanisms regulating TH-related signal transduction and development of therapeutic strategies [78,79,80].

In summary, TH signaling through receptors and integrin αvβ3 has important effects in the CNS because it modulates other processes in the brain such as dendritogenesis, neuritogenesis, and synaptic plasticity. It has been reported that nuclear TR activation and signaling pathways involving FAK-Akt and ERK1/2 are necessary for these processes [69]. Astrocytes effective interaction with neurons through these signaling pathways are necessary for a proper neuronal development and function, highlighting the relevance of coordinated cellular communication with other cells [81,82]. TRs modulate the expression of genes, but also activate signaling pathways non-genomic that play an important role in neurodevelopment and metabolism, which are of great importance in the face of NDDs and brain injuries [83,84,85]. Neurological sciences represent an opportunity to know these interactions to achieve a better use of TH as therapy.

## 5. The Roles of TH and HPT in NDDs

### 5.1. Thyroid Hormone and Alzheimer’s Disease

Thyroid hormones (THs) markedly influence AD through their effects on Aβ metabolism, tau phosphorylation, neuroinflammation, and oxidative stress mechanisms that are essential for maintaining synaptic homeostasis and neuronal survival. THs modulate amyloid and tau pathologies, control neuroinflammatory responses, and maintain the levels of oxidative stress. In summary, thyroid disturbance affects AD pathogenesis by inducing oxidative stress and mitochondrial dysfunction, which lead to cognitive decline.

Hypothyroidism and hyperthyroidism are associated with an increased risk of AD. Prevalence of hypothyroidism was much higher in AD patients than in controls [86]. The rate of clinical hypothyroidism and clinical hyperthyroidism were similar between AD patients and control subjects; however, alternative analyses revealed that overt hypothyroidism may protect against AD development and that hyperthyroidism may increase the risk of AD dementia [87]. These results imply that TH exerts a complicated effect on AD pathogenesis, which is supported by the Korean study on the prevalence of different thyroid disorders among AD patients [88,89]. Currently, evidence suggests that thyroid disturbance may increase AD risk by enhancing hypothyroidism and hyperthyroidism, which highlights the necessity of assessing thyroid function and promoting further studies on its effect on the progression of AD. A deviation in the levels of THs even within the so-called “normal” range is linked to the pathophysiology of AD. It has been demonstrated that patients present with changed FT4, and T3/T4 ratios, suggesting a disturbance in the conversion of T4 to T3, which may harmfully affect selected brain regions involved in AD [90]. In addition, reduced serum levels of T3 have been linked to an increased cerebrospinal amyloid burden and cognitive decay, underlining the important role of THs in Aβ-related pathology [91,92]. It has been reported that hypothyroidism is highly expressed in subjects with AD, indicating the presence of a positive loop in which hypothyroidism could be both a reason and an effect of the underlying pathological program of AD [23,93]. Additionally, autoimmune thyroid disease has been linked to greater amyloid and tau pathologies, regardless of hormone concentrations, indicating other potential pathways by which thyroid dysfunction may contribute to the progression of AD [94].

Hypothyroidism promotes AD progression by increasing amyloid-beta levels, facilitating tau phosphorylation, and impairing memory functions; meanwhile, AD pathology impairs TH metabolism, thereby forming a positive feedback loop [95,96]. In contrast, hyperthyroidism aggravated cognitive deficits and amyloid-beta accumulation by enhancing neuroinflammation and necroptosis (a programmed cell death process), suggesting that immune pathways may explain the increased AD risk due to TH disturbance. Irregularities in the levels of TH may promote amyloid-beta accumulation due to interference with the proper functioning of microglia. This is supported by the results obtained in murine models of Alzheimer’s, in which hypothyroidism results in detrimental effects on innate immune responses and an amplification of amyloid pathology [97]. In addition, THs are involved in tau pathology. The increased levels of free T3 and FT4 serum levels show a stronger association with the Aβ–tau burden, suggesting that THs may induce tau phosphorylation and aggregation in an Aβ-dependent manner [98]. The hyperphosphorylation of tau may be induced by impaired insulin signaling and increased activity of glycogen synthase kinase-3 beta (GSK-3β). The relationship between THs and neuroinflammation is also illustrated by the regulation of pyroptosis pathways. One of the most striking features of synaptic dysfunction is a lack of TH, which greatly modulates the expression of synaptic proteins and perturbs neurotransmission. Perturbed neurotransmission enhances cognitive impairments and neurodegeneration, forming a positive feedback loop in which thyroid deficiency induces neurodegeneration that, in turn, further impairs TH balance [97]. TH and AD pathology are not merely causally related; instead, they constitute a “vicious cycle” [23]. On the one hand, primary hypothyroidism can contribute to the onset and progression of AD through various pathways, such as inducing Aβ production, promoting tau protein hyperphosphorylation, triggering oxidative stress and endoplasmic reticulum stress, damaging mitochondrial function, and inhibiting BDNF [95]. On the other hand, the neurodegenerative lesions of AD itself, especially the accumulation of Aβ and phosphorylated tau protein, can in turn disrupt the metabolism, transport, and signaling of TH in the brain, resulting in secondary hypothyroidism within local brain tissue, which further exacerbates neuronal damage and cognitive decline [23,95]. This bidirectionally reinforcing relationship makes thyroid dysfunction both a risk factor for AD and possibly a result of its pathological progression. In total, these results highlight the important role of TH in the pathophysiology of AD, especially TH’s impact on amyloid-beta (Ab) metabolism and tau pathology; phosphorylation, neuroinflammation and oxidative stress are critical factors.

**Figure 2:**
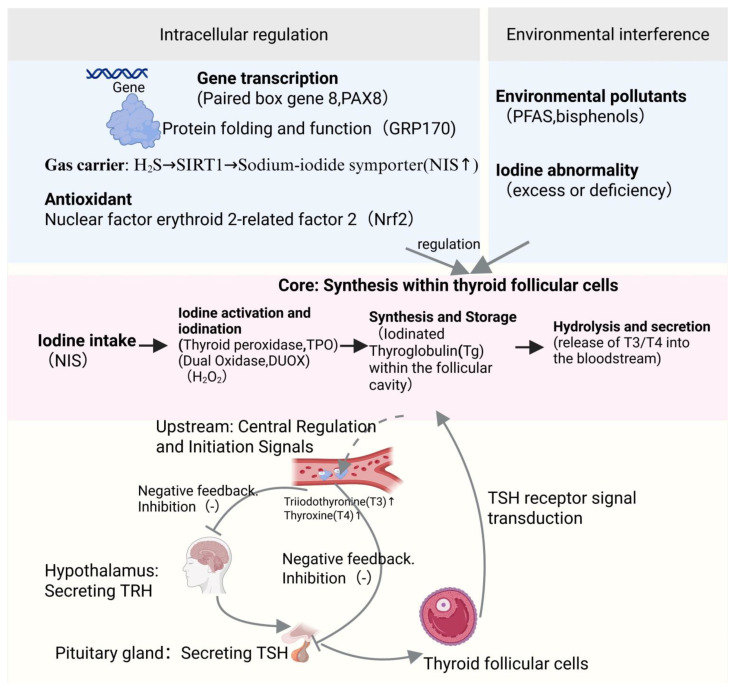
Mechanism of TH regulation in AD.

Interestingly, TH derivatives such as 3-iodothyronamine may correct AD pathogenesis by restoring synaptic functionality, which may provide new insights for the treatment of AD [99,100]. TH supplementation may correct the above-mentioned irregularities and enhance the activity of the anti-apoptotic protein Bcl-2, thus potentially diminishing tau-related pathology and neuronal apoptosis in models of AD [101]. For example, T3 supplementation may reduce the expression of microglial markers and pro-inflammatory cytokines in the hippocampus, and increase the expression of anti-apoptotic proteins, such as Bcl-2, to protect neurons [101,102]. For example, intranasal lithium treatment, which exerts effects on signaling related to THs, may effectively suppress pyroptosis and inflammatory responses in the cerebral environment of mouse models of AD [101,103]. Strategies targeting the modulation of TH signaling pathways may provide a promising way to slow down or prevent the progression of AD by recovering these vital protective effects (Figure 2).

### 5.2. Thyroid Hormone and Parkinson’s Disease

Thyroid hormones (THs) are responsible for modulating lipid metabolism and cerebral blood flow, which are of great importance in relation to PD. TSH may represent the link between peripheral metabolism and the CNS, which, in turn, modulates the energy balance and cellular homeostasis. Alterations in lipid metabolism are involved in NDDs, such as PD, potentially promoting neuroinflammation and increasing neuronal susceptibility [98]. Moreover, metabolic disturbances caused by hypothyroidism (such as obesity, increased cholesterol levels, and anemia) may complicate the pathology of PD by disturbing cerebral blood circulation and promoting oxidative stress [24]. Neuroimaging combined with microstructural analyses and molecular investigations have shown that there is dysfunction in the hypothalamus of PD patients. For instance, the mean kurtosis in particular hypothalamic subregions was decreased, and such changes were associated with TH levels and clinical manifestations. Such changes could be detected at the early stages of the disease and in patients with probable REM sleep behavior disorder (pRBD), which indicates that hypothalamic dysfunction may participate in the development of PD [96]. THs, namely T4 and T3, coordinate energy metabolism by modulating the metabolism of glucose, lipids, and proteins in different tissues. Dysregulation in the thyroid–liver axis has been implicated in the pathogenesis of metabolic disorders such as metabolic dysfunction-associated steatohepatitis (MASH) and early metabolic changes in PD [93]. In subjects diagnosed with MASH, increased serum levels of TSH and free triiodothyronine (FT3) and decreased FT4 levels independently predict the likelihood of developing MASH. These results suggest that an imbalance in TH could contribute to the sequestration of hepatic lipids and the promotion of inflammation [104].

It is known that there is a change in thyroid function in patients with PD; namely, the levels of FT3 are reduced, and TSH levels are increased, especially in patients who have cognitive impairment, which indicates that thyroid function may affect the severity of the disease [87]. In addition, thyroid dysfunctions encompassing hypothyroidism and hyperthyroidism are significantly associated with an increased risk of PD. Distinct associations with gender and age have been found. For instance, older males showed a stronger association with hypothyroidism, while hyperthyroidism was more common in younger females with PD [88]. TH abnormalities may help to explain PD and its motor as well as non-motor symptoms through their biological pathways. Thyroid dysfunction may mimic movement disorders such as PD, since hypothyroidism may present hypokinetic signs, and tremor is associated with hyperthyroidism. Therefore, it is possible to suggest that there is an association between thyroid hormonal imbalance and motor control [92]. In other words, for instance, in patients who are diagnosed with PD, different TH levels (particularly, a reduction in T3 and high TSH levels are associated with more severe motor symptoms and disease progression. So, it is evident that thyroid state has a great impact on the severity of PD [97]. Additionally, other non-motor symptoms, such as cognitive impairment, anxiety, and depression, have also been associated with TH variations in patients with PD. Thus, it has been demonstrated that low serum free T4 concentrations are significantly associated with cognitive decline in PD patients, and an integrated study of TSH, FT3, and free thyroxine may represent crucial biomarkers for cognitive impairment in elderly patients [87]. These clinical observations demonstrate the multiple and complex roles that TH assume in the symptomatology of PD, going beyond the well-known motor-related phenotypes. Serum TH and TH responsiveness markers are associated with components of the metabolic syndrome, such as insulin resistance and obesity. Moreover, FT3 and FT3/FT4 ratio are associated with different phenotypes of obesity, while TSH levels are associated with disturbances in glucose metabolism [105,106,107]. However, the results of Mendelian randomization studies have not found a causal relationship between thyroid dysfunction and the development of PD. Suggesting they may be contributing factors or modulators of disease progression rather than the primary etiological agents [91]. Despite the known correlations, Mendel randomization studies have not provided support for the causality between thyroid dysfunction and PD. This suggests that thyroid abnormalities may play the role of modifiers that may influence the severity and progression of PD rather than being the primary cause for the pathogenesis [91].

Therefore, it is essential to maintain a balance of TH as a basic pathophysiological mechanism involved in PD. Genetic studies have revealed associations between PD and thyroid disorders through shared susceptibility loci and immune pathways. It has been found that risk-associated loci affect the functions of thyroid and support the notion that immune dysregulation plays an important role in the coexistence of PD and thyroid disorders [89]. Furthermore, it has been found that the NR4A2 gene belonging to steroid-TH-retinoid receptor superfamily is correlated with the neurodegenerative process and movement disorder, indicating that TH signaling pathways may be involved in the pathogenesis of PD [95]. In addition, the autonomic denervation of the thyroid gland (shown by the reduced uptake in cardiac MIBG scintigraphy, an imaging method for the visualization of the thyroid gland by measuring the uptake of the radiopharmaceutical cardiac MIBG, which is a specific marker for sympathetic nerve function) is associated with the systemic metabolic changes induced by the PD, i.e., the TH levels were reduced, and the fatty acid β-oxidation regulated by the thyroid–liver axis was impaired. This autonomic dysfunction may break the normal feedback of the HPT axis and the release of TH (as shown in the extensive effects induced by the disease in the brain and body) [99,100]. Mechanisms of dopaminergic system–HPT axis cross talk. Regulatory mechanisms are disrupted in dopaminergic system–HPT axis, and the degree of disruption may be associated with the degree of PD and its motor complications. Autonomic denervation of the thyroid gland may be responsible for the dysfunction of the sympathetic nervous system to impair the secretion of TH. This dysfunction may cause metabolic disorders and unbalance, accelerating the course of PD [99,100]. Disruption of the autonomic–thyroid axis may impair β-oxidation of fatty acids in the liver. Illustration of the broken communication between the two organs represents an early biomarker for PD [99]. Moreover, genetic studies have shown the connected molecular pathways involved in the association between PD and thyroid disorders. It has been suggested that immune-related mechanisms play a critical role in the association between these two disorders, because many single nucleotide polymorphisms (SNPs) associated with susceptibility to PD also are associated with thyroid function, probably through immune-mediated pathways, so they may have an immunopathogenic basis [101]. The NR4A2 gene belongs to the steroid-TH-retinoid receptor superfamily and has been implicated in the pathogenesis of diverse human diseases, including neurodevelopmental, and neurodegenerative disorders such as PD [95]. The role of TH in PD not only affects metabolism and neural regulation but may also exacerbate pathology by interfering with basic cellular processes such as protein homeostasis and neuronal cell membrane function. Research indicates that the conformational stability of proteins is crucial for their function, and environmental factors such as pH changes can cause proteins to irreversibly transition from their native conformation to a denatured state [102]. Furthermore, TH is critical for regulating the physical properties of neuronal cell membranes. For example, the activity of membrane-bound enzymes (such as (Na+/K+)-ATPase) is highly dependent on the fluidity of their phospholipid environment, and components like cholesterol can alter this fluidity and significantly affect enzyme activity and temperature response curves [103]. TH imbalance may similarly change the lipid composition and fluidity of neuronal membranes, thereby affecting membrane protein function, signal transduction, and cellular homeostasis, providing a potential cellular biological basis for TH abnormalities exacerbating neuronal dysfunction in PD [103]. Impaired β-oxidation of fatty acids is an early manifestation of reduced β3-oxidation of fatty acids in PD, which is regulated by TH and thyroid deiodinase enzymes involved in modulating TH availability in the liver [94]. Moreover, microRNAs could epigenetically regulate the TH signaling in liver by targeting THRB and DIO1, which may contribute to the development of acquired hepatic resistance to TH in the progression of MASH. These molecular results further support the notion that the regulation of metabolism by TH is finely tuned in disease conditions [108]. Furthermore, intracellular TH metabolism, regulated by deiodinases and transcription factors such as FoxO1, is involved in cardiomyocyte hypertrophy and energy metabolism, which implies that TH signaling may also modulate metabolic tissues other than the liver [105,109]. There is reciprocal interaction between the regulation of dopamine and the activity of TH in PD [24,90].

Thyroid hormone (TH) and regulatory peptides are potential promising therapeutic options for PD due to their neuroprotective effects [86]. Pharmacological interventions to the dopaminergic system may maintain the balance of the HPT axis. For example, levodopa can normalize TSH and TH levels in patients with resistant PD and may be able to help to restore balance in the HPT axis and resolve PD-associated endocrine disorders [90]. Therefore, it is worth exploring biomarkers and potential treatments targeting the restoration of balance in the HPT axis. Furthermore, dopaminergic therapies (L-DOPA) used in the treatment of PD not only ameliorate motor symptoms but also modulate thyroid function by normalizing TSH and T3 hormone levels. These observations indicate the close interactions between neurological medications and endocrine regulation in the process of PD. However, based on the clinical evidence and molecular studies, it can be postulated that disturbances in the regulation of TH may affect various symptoms of PD and therefore represents an important target which needs to be corrected. Rectifying the TH levels imbalance may improve both motor and non-motor symptoms and possibly affect the progression of the disease. This indicates the need for further exploration for the relationships between thyroid function and PD as well as the development of individualized management. These results further support the possibility of developing new therapies targeting TH pathways to correct the disturbance in metabolism.TH replacement therapy with Levothyroxine is effective in hypothyroidism treatment. However, thyroid function-based individual dose-tailoring is necessary to prevent potential overtreatment or under treatment [110]. Selective THRB agonists, so called thyromimetics, may represent an innovative treatment option for hepatic diseases, such as hepatic steatosis or MASH. Recently, resmetirom was approved by the FDA [111,112]. In addition, the use of nanoparticle drug delivery systems improved the liver-targeting efficiency and the treatment efficacy of thyromimetics, which in turn facilitated the resolution of obesity-induced metabolic complications without impairing thyroid function overall [113].

### 5.3. TH and MS and Other Neurodegenerative Diseases

Thyroid hormone (TH) level plays a critical role in both demyelination and remyelination process. Demyelination is more pronounced in hypothyroid rats than in euthyroid controls, suggesting that THs have a protective effect against demyelination [95,96,99]. Based on the regulation of immune-related genes by TH and their impact on the neuroinflammatory environment, TH deficiency may exacerbate neuroinflammation and disease progression by promoting the expansion of pathogenic immune subsets such as Eomesodermin+ helper T cells (Eomes+ Th) [92]. This indicates that TH signaling plays an important role in maintaining immune homeostasis in the CNS, and its dysregulation may contribute to the development of neurodegenerative lesions. Therefore, TH deficiency may promote MS and other NDDs by disrupting the homeostasis of the neuro-immune-metabolic network, including affecting metal ion balance and the oxidative stress and inflammatory pathways they mediate [97]. Deficiency of TH is increasingly being recognized to contribute to the development of NDDs such as AD, PD, Huntington’s disease (HD), and MS. This is due to the important roles that THs play during brain maturation and neuronal protection. With respect to HD, the absence of THs may prolong disease progression due to increased neuronal vulnerability, although there is limited research studying THs and its pathophysiology with HD [101]. The pathological correlation between TH deficiency and MS is further supported by observations indicating that low TH levels result in incomplete remyelination and persistent neurological defects [102]. In addition to directly affecting the CNS, alterations in TH functions may also worsen systemic inflammation and immune dysregulation, which may facilitate the progression of diseases such as MS, where immune-mediated processes play a major role. Deficiency in THs induce NDDs such as Huntington’s and MS by neuronal survival and myelin integrity, which involves aspects of cellular energy metabolism and oxidative stress and immune dysregulation. TH is crucial for maintaining immune balance, and its deficiency may lead to the expansion of pathogenic T helper cell subsets. suggesting that TH may influence the progression of various NDDs by regulating such cells. These findings indicate that TH deficiency promotes neurodegenerative lesions through multiple mechanisms, including direct action on glial cells, indirect effects on the function of specific immune cells, and disruption of the homeostasis of the neuro-immune-metabolic network.

Thyroid hormone (TH) affect the myelination process in the CNS by interaction with nuclear receptors. TH receptor have been implicated in oligodendrocyte precursor cell differentiation into mature oligodendrocytes that produce or re-myelinate myelin and oligodendrocytes in MS [86]. TH receptors activation leads to the up-regulation of transcription of genes encoding myelin proteins and facilitate myelin protein synthesis and maintenance of myelin sheath [87,88,89]. The molecular mechanisms underlying Sob-AM2 actions include both genomic effects on myelin-related genes and non-genomic signaling pathways. TH may act through the integrin receptors on oligodendroglial cells and induce specific signaling cascades that promote oligodendrocyte maturation and myelin sheath development [90]. These pathways may be involved in integrating specific extracellular signals and coupling them with intracellular metabolic activity and growth factors, which in turn promote differentiation of OPCs into myelinating OLs during the remyelination process. Additionally, TH has been shown to regulate many immune-related genes, including TREM2, and therefore suggests that when TRs are activated there are significant changes in the neuroinflammatory environment that likely plays a role in the pathophysiology of demyelinating diseases [91]. In addition, THs are involved in maintaining the balance of trace elements (such as zinc), and zinc deficiency disrupts the balance among Th1/Th2/Th17 cells, which is considered one of the pathogenic mechanisms of MS [97]. THs are required for the maturation of oligodendrocytes and myelin synthesis in MS. TH receptors mediate remyelination and maintain myelin integrity; hence TH receptors may serve as potential targets for remyelination in MS and other disorders [18,21]. THs are essential for the expression of genes involved in neurogenesis and energy metabolism; any abnormalities in TH levels will lead to NDDs. Furthermore, THs play a major role in regulating systemic metabolic functions such as fatty acid β-oxidation. Investigators have implicated TRH as a promoter of TH secretion [93]. Neuroprotective effects of TRH have been demonstrated through its ability to activate the PI3K/AKT pathway and protect neurons against excitotoxic conditions [28]. In addition to the effects on classic pathways such as myelination and cellular energy metabolism mentioned above, the mechanism by which TH deficiency promotes other NDDs also includes its role in fine-tuning the central nervous immune microenvironment. Additionally, TH is involved in maintaining the homeostasis of trace elements (such as zinc), and zinc deficiency can disrupt the balance between Th1/Th2/Th17 cells, which is believed to contribute to the pathogenesis of MS [97].

Based on this knowledge, research has attempted to mimic this molecular event by applying T3 in combination with apo-transferrin and found that this combination has synergistic effects on oligodendrocyte maturation and remyelination in vivo. The beneficial effects of this combination were markedly reduced by administration of TH receptor antagonists, which significantly promote oligodendrocyte maturation and in vivo remyelination [100]. Selective TRβ agonist Sob-AM2 promoted remyelination in oligodendrocytes with little or no peripheral side effects attributable to TH, as assessed by enhanced myelin regeneration in demyelinated murine models [16]. Clinically, from a preclinical standpoint, and even from initial clinical findings, there is reason to be optimistic that targeting TRs may be a viable approach to treat demyelinating diseases. Success in large animal models of disease has shown thyromimetic agents to promote remyelination and help recover function in the setting of significant CNS demyelination, opening up a promising opportunity to translate these findings into treatment for human MS [18]. Overall, these observations have set the stage for success with TR agonists. While these represent significant opportunities, there is a clear gap in the clinical trials space that highlights the need for careful assessment of the safety profiles of TR agonists, given the varied systemic effects of TH, including on the cardiovascular system, metabolic effects, and brain physiology [98]. Initial clinical findings have shown some degree of efficacy and tolerability, but larger and more integrated studies are needed to validate these results. Nonetheless, the overall body of work has provided strong evidence that TH signaling, through nuclear receptors, plays an essential role in oligodendrocyte differentiation, synthesis of myelin proteins, and remyelination, thereby positioning TRs as promising therapeutic targets for demyelinating diseases Investigations on modulation of THs and its effects on NDDs have gained considerable interest. At present, investigators are evaluating the effects of TRH analogs with improved pharmacokinetic properties to determine whether they can improve cognitive function, slow down the progression of NDDs and reduce significant fluctuations in the systemic TH levels [94]. Furthermore, thyromimetic agents such as sobetirome and new diphenyl-methane derivatives have been shown to be effective in stabilizing transthyretin amyloidosis, a pathologic process involved in neurodegeneration, highlighting the potential of TH analogs and their substantial therapeutic benefit [108]. Therefore, novel therapies targeting the TH pathway, such as centrally selective TH receptor β agonists (e.g., sobetirome and its prodrug Sob-AM2), which have shown significant myelin repair potential in preclinical models [16,106], have become promising therapeutic strategies.

## 6. The Cellular and Molecular Signalling Mechanisms of TH and HPT in Neuroprotection and Anti-Neuroinflammatory Effects

### 6.1. Neuroprotective Mechanisms of TRH

**Figure 3:**
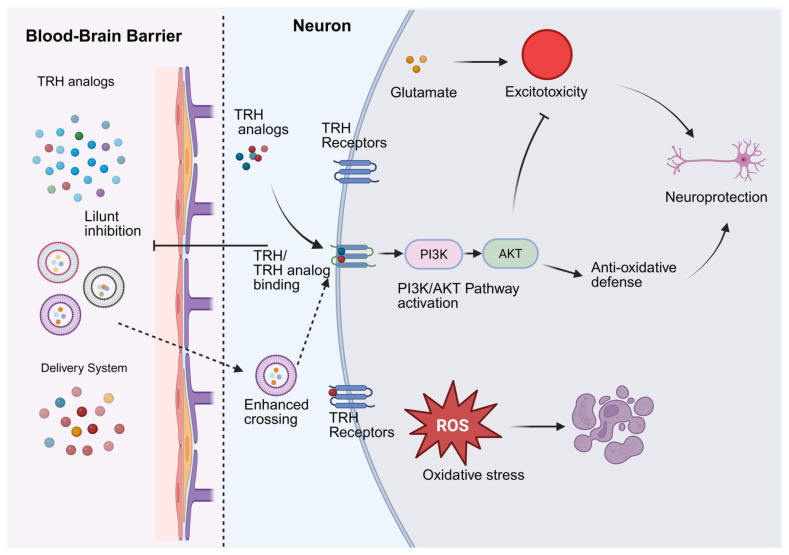
Thyroid hormones (THs) are key neuroprotective and anti-neuroinflammatory signaling mechanisms.

TRH has been widely studied as an important hormone existing in the HPT axis, which can promote TSH and prolactin secretion. In addition to the endocrine function, TRH has been reported to be neuroprotective in NDDs and brain injuries (traumatic brain injury, TBI). This neuroprotective effect of TRH may be associated with the PI3K/AKT signaling pathway, which mediates the suppression of glutamate-induced excitotoxicity [28,114]. In addition, the neuroprotective effect of TRH analogs may be related to the protection against oxidative stress. However, TRH analogs cannot be widely used in clinic because of the limited penetration of TRH analogs into the brain via the blood–brain barrier [115,116]. Thus, it is essential to study TRH analogs and corresponding delivery systems for the treatment of NDDs and brain injuries. This highlights the importance of studying receptor signaling in clinical neurology [117,118] (Figure 3).

### 6.2. TH and Neural Stem Cells (NSCs) Function Regulation

Thyroid hormones (THs), either T3 or T4, play a positive modulatory role in the regulation of NSCs that give rise to the brain and are involved in brain repair. THs are mainly present in the neurogenic areas of the brain such as the SVZ and the SGZ. In these regions, T3 promotes the commitment of NSCs to neuronal fates and modulates the differentiation of OPCs, which are important for oligodendrocyte myelination [119,120]. The molecular mechanisms underlying these actions are partly mediated through genomic pathways, such as the translocation of TH receptors into the nucleus, and partly through non-genomic pathways that regulate mitochondrial activities [121], which affect the self-organization of progenitor cells at different developmental stages. Transcriptomic analysis in *Xenopus laevis* has shown that signaling through the TH pathway is essential for regulating gene expression in NSCs and progenitor cells. T3 exposure results in the upregulation of genes involved in the biogenesis and functions of ribosomes, translation, mitochondria, and proteasomes, which are important for the activation of NSCs and neurogenesis [122].

In TBI, MS, and other NDDs, NSCs provide valuable insights into repair processes. Data support the notion that THs induce the proliferation and differentiation of NSCs toward neurons and oligodendrocytes to promote remyelination and neuronal regeneration [123]. T3 attenuates neuronal mortality after TBI through mitophagy and enhances neurogenesis, suggesting that T3 has dual protective and regenerative effects [124]. The transport and localized activation of THs within neurogenic niches are regulated by MCT8 and DIO2, which are involved in NSC signaling. Knockout of these proteins results in defects in neurogliogenesis and olfactory functions, highlighting the importance of TH metabolism during brain repair [125,126]. Ratios of TH concentrations at critical periods of development, such as those caused by hypothyroidism or PFAS-induced disruption of TH signaling, can inhibit NSC growth and differentiation, leading to lifelong impairments in neurogenesis and oligodendrogenesis. Specifically, PFAS disrupts oligodendrogenesis and remyelination. T3 may rescue these effects, suggesting that modulation of TH signaling could provide protection against the environmental toxin PFAS [119,127,128]. THs regulate NSCs, specifically tanycytes, which display stem cell-like properties. Decreases in the expression of neural cell adhesion molecules such as NrCAM, are associated with decreased tanycyte populations and related markers, suggesting that TH signaling regulates the maintenance and differentiation of NSCs [129]. In addition to the mechanisms described above, THs exert a large degree of control over NSCs through interactions with other signaling pathways, such as Sonic Hedgehog, The TRIP6-YAP-SHH axis is particularly important for the maintenance and differentiation of NSCs in the postnatal SVZ [130]. Synthetic agonists modulating TRβ1 can increase the differentiation of OPCs in inflammatory contexts, suggesting new potential therapies for demyelinating diseases [131]. We identified TH transporters and receptors within human neural progenitor cells and neurons using human cerebral organoid models. These data support the idea that TH signaling plays a role in cortical development and neurogenesis, which is useful for understanding the mechanisms underlying TH-related neurodevelopmental disorders [132,133,134].

Despite achieving biochemical euthyroidism, many patients still experience lingering symptoms like cognitive impairment and fatigue. This finding implies a potential shortcoming in TH signaling within the CNS [135,136]. Levothyroxine monotherapy results in an increase in serum free T4; however, it may cause a decrease in serum free T3 due to impaired conversion function, which may be detrimental to neuropsychiatric symptoms [137,138]. For some patients, a combination approach with liothyronine (LT3) in conjunction with LT4 may improve quality of life and reduce symptoms, despite a paucity of information regarding the long-term safety of this combination therapy [135,139]. Variability in genetic polymorphisms of deiodinase enzymes may benefit from individualized consideration regarding TH replacement therapies [137]. Hypothyroidism worsens cognitive decline and neuropsychiatric symptoms in patients with NDDs, TH replacement therapy (THRT) partially eliminates these problems, but cannot eliminate them altogether, highlighting the shortcomings of our treatment modalities [136]. The use of extended LT4 therapy risks subclinical hyperthyroidism, which increases cardiovascular risks in the elderly population and complicates management regimens [140,141]. Both under- and over-treatment are detrimental to patient outcomes, and careful consideration of THRT dosage and monitoring is imperative [141,142]. In special populations, like pregnant women, careful titration of THRT is necessary to maximize neurodevelopmental outcomes for both mother and child [142,143]. Novel treatment modalities utilizing thyroid extracellular matrix hydrogels are being investigated to overcome the limitations of hormone replacement therapy and restore thyroid function, with potential advantages for the treatment of NDDs [144,145]. TH replacement therapy successfully normalizes TH levels but struggles to restore tissue function, particularly in the brain. Patient variability in response highlights an opportunity for improvement and calls for further research and advanced treatment strategies for NDDs associated with hypothyroidism.

Thyroid hormones (THs) are closely related to the pathological processes of various NDDs, and their changes are associated with key pathological markers. They participate in disease progression by affecting specific neural pathways [146,147]. Abnormal thyroid function can alter the processing of β-amyloid precursor protein [146], and the neurotoxicity mediated by thyroid hormone response proteins suggests that TH signaling may intersect with amyloid pathology [146]. In PD, TH levels are significantly associated with cognitive dysfunction1; the increased co-expression of dopamine transporters and tyrosine hydroxylase in peripheral immune cells suggests a common regulatory abnormality [148]. In animal models, promoting TH signaling can increase the number of dopaminergic neurons in the substantia nigra and dopamine levels, improving motor function [149], indicating that TH supports the survival and function of dopaminergic neurons, and its dysregulation may participate in pathology by affecting dopamine synthesis and transmission. In MS, the role of TH focuses on myelination and oligodendrocyte function, and its promotion of oligodendrocyte maturation and myelination depends on the activation of transcription and metabolic pathways within the CNS [150]. The metabolic dysfunction of oligodendrocytes and the dysregulation of TH-dependent pathways in MS constitute one of the key mechanisms driving demyelination and neurodegeneration; thus, TH dysregulation is mainly associated with myelin pathology. In addition, the frequency of Eomesodermin-expressing Th cells is increased in MS patients, and these cells may also play a role in other NDDs [151].

### 6.3. The Expression of TH in the Brain Region of NDDs

The expression of TH in the CNS and metabolic alterations are important components of the pathophysiological mechanisms of various NDDs, showing significant regional specificity in different diseases. In AD, pathological changes exceed the variations in serum TH profiles [90]. Studies have found that local hypothyroidism occurs in the hippocampus (a key brain region related to memory) of AD model mice, with the mechanism being the specific downregulation of deiodinase type 2 that converts thyroxine to active triiodothyronine. This local hormone deficiency in brain tissue can occur earlier than changes in peripheral blood indicators. It can also inhibit the innate immune response of microglia, thereby exacerbating disease progression [97].

In PD research, clinical observations suggest that TH levels are related to the degree of cognitive impairment in PD patients [146]. More notably, studies have found that the expression of dopamine transporters and tyrosine hydroxylase is significantly upregulated in peripheral blood mononuclear cells of PD patients, and this phenomenon is replicated in PD animal models, suggesting that central lesions in the nigrostriatal dopaminergic pathway may trigger compensatory changes in peripheral immune markers [148].

For MS, the role of TH signaling in promoting myelin regeneration is a research focus. TH and CNS-selective agonists can drive oligodendrocyte precursor cell differentiation and effectively promote remyelination in MS animal models [152,153]. In experimental autoimmune encephalomyelitis models, supplementation with triiodothyronine has been confirmed to protect myelin, maintain nerve conduction, and preserve axonal integrity [154]. However, systemic administration of TH in chronic demyelination models may inhibit endogenous repair, while central selective TH receptor agonists show better therapeutic potential [153].

In HD, lesions are highly concentrated in the striatum. Research reveals that the expression of a key TH binding protein—μ-crystallin—is downregulated in the striatum of HD animal models [155]. This protein is specifically highly expressed in the striatum, and its absence enhances the susceptibility of striatal neurons to the toxicity of mutant huntingtin protein, indicating that TH-related local metabolic dysregulation may be one of the core factors leading to the selective vulnerability of this brain region [156].

## 7. Research and Application of Thyroid Hormones and Related Derivative Drugs

### 7.1. Therapeutic Perspectives and Clinical Potential

Thyroid hormone(TH) analogs, like sobetirome, which are selective agonists of TRβ, possess a remarkable potential for the treatment of neurodegenerative disorders due to their ability to promote neuronal wellbeing and myelin repair with a better safety profile than natural thyronines [16]. Interestingly, recent studies have revealed that TH analogs can stabilize transthyretin (TTR), a protein essential for TH transport and responsible for neurodegenerative amyloidosis, by preventing TTR aggregation, making them promising multi-target drugs for the treatment of the hereditary amyloidosis, familial amyloid polyneuropathy and even AD. In addition, sobetirome analogs are expected to provide therapeutic benefits for genetic diseases, such as Allan–Herndon–Dudley syndrome, which is caused by mutations in the MCT8 gene that lead to defective TH transport to the brain and neurological deficits by accumulating thyroid signaling in the CNS (e.g., Sob-AM2 can be used for brain accumulation and myelin repair in NDDs) [157]. In addition, sobetirome displays certain anti-inflammatory activities that may be beneficial for neurodegenerative disorders. For instance, TH analogs can modulate macrophage polarization and suppress M1 phenotype, an activated macrophage state that is involved in inflammation (TRβ1-activated macrophages), which may represent a potential mechanism for immunomodulation in neuroinflammation [158]. With the development of molecular biology, receptor-selective compounds are being developed to treat CNS disorders while alleviating side effects in distant organs. Recently, TH analog, resmetirom, approved for metabolic disorders, is an example [159]. Innovative TH analogs like sobetirome may represent novel therapeutic approaches for the treatment of NDDs by regulating metabolic process, preventing amyloid aggregation, promoting remyelination and reducing inflammation while bypassing defective transport system and activating beneficial receptors. However, the in vivo effectiveness and safety of TH analogs should still be further investigated [157].

Thyrotropin-releasing hormone (TRH), being a tripeptide, plays a key role in regulating the HPT axis and also exhibits neuroprotective properties; however, its clinical use is limited due to poor absorption and rapid degradation [160]. Therefore, these characteristics have motivated numerous studies on novel strategies for avoiding the above-mentioned delivery approaches and the formulation of TRH analogs that retain biological activity but overcome these limitations. It has been reported that TRH could be effectively used to bypass the blood–brain barrier, which improves the CNS accessibility [161]. Meanwhile, the application of biodegradable polymer-based nanoparticles can provide high drug loading and controlled release, which are critical for the above-mentioned therapeutic effect. Subsequently, by employing intranasal refillable atomizers, these nanoparticles were effectively delivered to the olfactory neuroepithelium, which improved the cerebral delivery while reducing the systemic degradation. For the above-mentioned TRH-loaded nanoparticles, in vivo safety evaluations using cynomolgus monkeys and rats demonstrated that there was no obvious systemic adverse reaction and no apparent endocrine disturbance, and the TSH and prolactin levels were well maintained. There was slight damage to the nasal tissue. Overall, these results demonstrated the safety and biocompatibility of the above-mentioned nanoparticles [162]. No apparent endocrine disturbance was noteworthy, given that TRH is involved in the HPT axis and there is a potential risk of inducing unexpected hormonal effects. Sophisticated delivery system: lipid-core polymeric nanocapsules: improve the bioavailability and effectiveness of TRH by enhancing the encapsulation and delaying the release rate of TRH to achieve the best pharmacodynamics [163]. Chemical permeation enhancers such as carveol and borneol promote the diffusion of peptides into the dermis. However, the CNS is still favored for intranasal administration because it is directly accessible and has low systemic exposure [164]. The analogs of TRH such as taltirelin have good pharmacokinetics and enhance receptor affinity. The neuronal activation is enhanced, and new possibilities for the pharmaceutical use of taltirelin for OB APA have been shown [165]. AAV vectors expressing TRH-related peptides were transfected into the brain, and the expression and secretion of peptides in the CNS were increased, and the problems related to effective and targeted delivery were solved [166]. TRH can activate the PI3K/AKT signaling pathway for neuroprotection. Therefore, accurate administration of TRH is necessary for neuroprotective agents [28]. TRH is an important neuropeptide with a wide range of physiological activities. Many advances have been made in the development of TRH analogs and means of enhancing the blood–brain barrier permeability and resisting degradation, which enhance the neuroprotective effect by improving therapeutic efficacy and maintaining therapeutic time. Therefore, TRH is expected to play a more important role in amplifying therapeutic efficacy and reducing side effects by developing selective TRH analogs involving β-arrestin2 in TRH receptor signaling [167]. The recent progress in administering TRH via the intranasal biodegradable nanoparticles system has enhanced its safety and bioavailability within the CNS. The new TRH-based therapy for neurodegenerative and neuropsychiatric disorders will be realized based on the ongoing investigations in the delivery system and TRH receptor signaling.

Search strategies for therapeutic approaches against neurodegenerative disorders are increasingly embracing the concept of combinatorial therapies. However, THs are not able to resolve the neuropsychiatric or cognitive impairments present in hypothyroidism or NDDs [20,104]. Magnesium threonate (MgT) readily crosses the blood–brain barrier, increases brain magnesium content, and exhibits neuroprotective properties in AD through the reduction in Ab accumulation and increase in BDNF levels [104]. MgT reduces Ab concentrations in hypothyroid models lacking TH, suggesting that it may improve the efficacy of combination therapy with THs [104]. The use of combined treatment strategies combining THs and MgT may provide neuroprotective advantages owing to their respective hormonal and neuroprotective deficiencies, as well as those resulting from neuroinflammation. Furthermore, TRH has been shown to be effective against glutamate-induced neurotoxicity and may also provide neuroprotective advantages when combined with THs [28,160]. Combinations of THs and agents such as MgT or TRH analogs hold promise for the treatment of NDDs; however, the pharmacokinetics and interactions of these substances should be carefully investigated to ensure treatment safety and efficacy. Future clinical trials targeting TH treatments for NDDs should utilize advanced study designs that take into account the complexities of these diseases, and participants should be stratified according to thyroid function, genetics, and other relevant biomarkers [20,168]. Precision medicine strategies using omics approaches can help identify patients who may benefit from TH treatments or other integrative therapies involving neuroprotective agents, such as magnesium L-threonate or TRH analogs [168,169]. Neuroimaging and thyroid–liver axis-related biomarkers, which reflect the complex regulatory network involved in TH metabolism and systemic homeostasis, can provide more accurate therapeutic evaluations and assessments of disease progression [168,169]. Novel drug formulations, such as intranasal administration of TRH analogs, may enhance therapeutic efficacy by addressing issues related to blood–brain barrier permeability and agent degradation [160]. The design of trials should include assessments of cognitive, motor, and neuropsychiatric parameters, as well as the use of biomarkers for evaluating treatments. Furthermore, adaptive approaches may facilitate the rapid identification of effective therapies. The synthesis of knowledge regarding TH and neuroprotective approaches may eventually lead to personalized treatments for NDDs.

### 7.2. Comprehensive Strategies to Improve TRH Delivery Systems, Including Targeted Delivery and Safety Considerations

In conclusion, the clinical efficacy of TRH and its analogs depends on the effectiveness of the delivery system, and the core challenge is overcoming biological barriers to achieve effective delivery to the target site (Table S1). The enhancement strategies mainly include: overcoming the blood–brain barrier through chemical modification or RNA interference to enhance delivery to the CNS [155,170]; achieving direct and sustained drug delivery to the brain through local implantation [171]; utilizing nanocarriers to improve the physicochemical properties and pharmacokinetics of drugs for controlled release [163], and improving oral bioavailability via formulation design [172]. However, safety evaluation is crucial, and any delivery system must be rigorously evaluated for long-term safety [173]. In the future, it is necessary to combine multiple strategies to improve delivery efficiency while deeply understanding the therapeutic mechanisms and ensuring safety to maximize the clinical potential of TRH.

## 8. Conclusions

Thyroid hormones (THs) are essential for normal development of the nervous system and have also been demonstrated to be intimately involved in the pathology of neurodegenerative disorders. The level of active TH T3/FT3 is reduced, and this hormone plays an important role in the pathophysiology and clinical progression of PD [146,174]. Monitoring these hormone levels is useful for assessing patients’ prognosis. Future treatment strategies may include developing TH analogs specific to the CNS [175], regulating hormone pathways in specific populations (such as RTHβ patients) [176], and targeting downstream pathogenic pathways related to TH (such as oxidative stress) [177]. Thyroid hormone analogs have entered clinical applications, such as Resmetirom, which has been approved for the treatment of MASH, providing a precedent for neurological applications [175]. Treatments for genetic disorders, such as Triac, have shown benefits in RTHβ patients, demonstrating the feasibility of targeting abnormal pathways to treat neurological symptoms [175,176]. Basic research has revealed new targets, such as YOD1, which exacerbate dopamine neuron damage via the PKM2-Nrf2 axis, potentially providing neuroprotective strategies for PD [177]. However, several unresolved issues remain: the specific mechanisms of thyroid hormone neurotoxicity, particularly the interactions of THRP with proteins like c-Abl, are still unclear [146]; differences in sensitivity to hormonal fluctuations among different populations pose challenges for personalized treatment [174]; and there is a lack of systematic research on how to integrate upstream hormone regulation with downstream pathway therapies. Several limitations also exist: most evidence comes from observational studies [174], making it difficult to establish causality; some findings, such as specific gene mutations, are based solely on case reports [176], requiring validation of their generalizability; many targets and mechanisms are derived from model studies, necessitating experimental evaluation for clinical translation [177]; and the development of centrally selective analogs faces challenges in delivery and tissue selectivity.

## Data Availability

Data sharing not applicable to this article as no datasets were generated or analyzed during the current study.

## Supplementary Materials

The following supporting information can be downloaded at: https://www.mdpi.com/article/10.3390/brainsci16020229/s1, Table S1: TH-related therapeutic agents and their applications in NDDs.
