# Supplementary material for: Emerging Roles, Mechanisms, and Therapeutic Potential of Thyroid Hormones in Neurodegenerative Diseases: A Review
Source: Brain Sci. 2026 Feb 14;16(2):229. doi: 10.3390/brainsci16020229 (PMC12938659; doi:10.3390/brainsci16020229)
Supplement: Supplementary file 1 [file brainsci-16-00229-s001.zip › brainsci-4071202-supplementary.pdf]

Table S1. TH-related therapeutic agents and their applications in NDDs

Category/Agent	Key Characteristics/Delivery Strategies	Therapeutic Potential/Main Advantages
**TH Analogs**		
Sobetirome & analogs (e.g., Sob-AM2)	Selective TH receptor beta (TRβ) agonists.	Promote neuronal health and myelin repair with a superior safety profile compared to natural thyronines. Potential for treating neurodegenerative disorders (e.g., multiple sclerosis) and Allan-Herndon-Dudley syndrome (targeting cerebral TH transport defects).
TTR-stabilizing TH analogs	Stabilize transthyretin (TTR), inhibiting its aggregation.	Function as multi-target agents for hereditary amyloidosis, familial amyloid polyneuropathy, and Alzheimer's disease.
Resmetirom	A TH analog approved for metabolic disorders.	Serves as a paradigm for developing CNS-targeted selective compounds designed to minimize peripheral side effects.
**TRH and Delivery Strategies**		
TRH (Thyrotropin-releasing hormone)	A tripeptide with poor absorption and rapid degradation.	Possesses neuroprotective properties, activating the PI3K/AKT signaling pathway.
TRH-loaded nanoparticle delivery systems	Employ biodegradable polymer-based nanoparticles delivered via intranasal refillable atomizers.	Enhance brain delivery, reduce systemic degradation, enable controlled release, and demonstrate favorable safety and biocompatibility.
Chemical permeation enhancers (e.g., Carveol, Borneol)	Facilitate peptide permeation.	Improve drug diffusion, particularly for intranasal administration to minimize systemic exposure.

TRH analogs (e.g., Taltirelin)	Feature improved pharmacokinetics and receptor affinity.	Enhance neuronal activation, offering new pharmaceutical possibilities.
AAV vectors expressing TRH-related peptides	Enable targeted gene delivery to the brain.	Address challenges in effective and targeted delivery, increasing peptide expression and secretion within the CNS.
**Combination Therapeutic Strategies**		
THs + Magnesium L-threonate (MgT)	MgT crosses the blood-brain barrier, elevates brain magnesium, reduces Aβ accumulation, and increases BDNF levels.	Combination may confer synergistic neuroprotection, compensating for respective hormonal and neuroprotective deficiencies and targeting neuroinflammation.
THs + TRH (or its analogs)	TRH counteracts glutamate-induced neurotoxicity.	Co-administration holds promise for enhanced neuroprotection, warranting further investigation into pharmacokinetics and interactions.
